# Lipocalin-2 and insulin as new biomarkers of alopecia areata

**DOI:** 10.1371/journal.pone.0268086

**Published:** 2022-05-31

**Authors:** Anna Waśkiel-Burnat, Anna Niemczyk, Paulina Chmielińska, Marta Muszel, Michał Zaremba, Adriana Rakowska, Małgorzata Olszewska, Lidia Rudnicka

**Affiliations:** Department of Dermatology, Medical University of Warsaw, Warsaw, Poland; University of Alberta, CANADA

## Abstract

Lipocalin-2 and visfatin are proinflammatory adipokines involved in the regulation of glucose homeostasis. Their role has been described in numerous inflammatory skin diseases such as atopic dermatitis and psoriasis. Recently, an increased prevalence of metabolic abnormalities has been reported in patients with alopecia areata. The aim of the study is to determine the serum levels of lipocalin-2 and visfatin in patients with alopecia areata in comparison with healthy controls. Moreover, the serum levels of total cholesterol, low-density lipoprotein cholesterol (LDL-cholesterol), high-density lipoprotein cholesterol (HDL-cholesterol), triglycerides, fasting glucose, insulin, c-peptide, and homeostasis model assessment for insulin resistance (HOMA-IR) were evaluated. Fifty-two patients with alopecia areata and 17 control subjects were enrolled in the study. The serum levels of lipocalin-2 [mean ± standard deviation, SD: 224.55 ± 53.58 ng/ml vs. 188.64 ± 44.75, p = 0.01], insulin [median (interquartile range, IQR): 6.85 (4.7–9.8) μIU/ml vs. 4.5 (3.5–6.6), p<0.05], c-peptide [median (IQR): 1.63 (1.23–2.36) ng/ml vs. 1.37 (1.1–1.58), p<0.05)], and HOMA-IR [median (IQR): 1.44 (0.98–2.15) vs. 0.92 (0.79–1.44), p<0.05) were significantly higher in patients with alopecia areata compared to the controls. The serum concentration of insulin and HOMA-IR correlated with the number of hair loss episodes (r = 0.300, p<0.05 and r = 0.322, p<0.05, respectively). Moreover, a positive correlation occurred between insulin, HOMA-IR, c-peptide and BMI (r = 0.436, p <0.05; r = 0.384, p<0.05 and r = 0.450, p<0.05, respectively). In conclusion, lipocalin-2 and insulin may serve as biomarkers for alopecia areata. Further studies are needed to evaluate the role of insulin as a prognostic factor in alopecia areata.

## Introduction

White adipose tissue plays a crucial role in lipid storage, thermoregulation, mechanical organ protection and the regulation of energy homeostasis [1]. It also has a central role in the regulation of immune responses; thus, it is considered as an immunometabolic system [1]. White adipose tissue produces various hormones and bioactive molecules called adipokines [2]. Currently, more than 600 adipokines presenting various biological functions/features in the human body have been described [1]. They are characterized by pro- and anti-inflammatory properties. The up-regulation of pro-inflammatory adipokines induces a chronic, low-grade inflammatory state and contributes to metabolic dysfunction. The role of anti-inflammatory adipokines in these processes is under investigation [1]. Adipokines were also described as the active regulators of cutaneous inflammatory processes. Their role was indicated in numerous dermatological conditions such as psoriasis, atopic dermatitis, acne, rosacea, and melanoma [1].

Alopecia areata is an autoimmune form of non-scarring hair loss that may affect any hair-bearing area [3]. The pathogenesis of the disease has not been fully described. Numerous studies indicated that alopecia areata is associated with systemic autoimmune activation implying significantly elevated serum levels of Th1 (IL-1β, IL-2, IL-12, TNF-α, and IFN-γ), Th2 (IL-4, IL-10, IL-13, IL-25, IL-31) and Th17 cytokines (e.g. IL-17A) [4]. Recently, a higher prevalence of metabolic disorders has been described in patients with alopecia areata compared to healthy individuals [5]. Moreover, an impaired level of adiponectin and leptin was reported in patients with alopecia areata in comparison with the control group [6].

Lipocalin-2 and visfatin are adipokines involved in the regulation of glucose homeostasis as well as systemic inflammation [7, 8]. Their role in numerous inflammatory diseases such as rheumatoid arthritis, psoriasis or atopic dermatitis has been described [8–12]. To date, data considering the role of lipocalin-2 and visfatin in alopecia areata is limited.

### Objective

The aim of the study was: (1) to evaluate the serum levels of lipocalin-2 and visfatin in patients with alopecia areata in comparison with healthy controls; (2) to determine the serum levels of total cholesterol, low-density lipoprotein cholesterol (LDL-cholesterol), high-density lipoprotein cholesterol (HDL-cholesterol), triglycerides, fasting glucose, insulin, c-peptide, and homeostasis model assessment for insulin resistance (HOMA-IR) in patients with alopecia areata compared to healthy controls.

## Patients and methods

### Patients

All eligible patients diagnosed with alopecia areata consulted in our department between February 2021 and May 2021 were screened as regards inclusion in this study. The inclusion criteria were as follows: patients over 18 years of age, the diagnosis of alopecia areata, no systemic treatment for alopecia areata in the previous three months. The exclusion criteria were: autoimmune diseases other than alopecia areata, malignancy, pregnancy and breastfeeding, and prior hyperlipidemia, diabetes mellitus, or insulin resistance. The control group comprised healthy individuals matched for age, gender and body mass index (BMI) with the study group. The same exclusion criteria referred to control group subjects.

Demographic data and clinical variables such as age, gender, weight, and height were collected in all individuals. BMI was calculated as: weight (kg) / height^2^ (m). Additionally, data concerning the age when the first episode of hair loss occurred, the number and duration of the present episode of hair loss were recorded in patients with alopecia areata. The severity of hair loss was assessed with the severity of alopecia tool (SALT) and graded as S1 to S5 according to the guidelines of the National Alopecia Areata Foundation [13] as follows: S0, no hair loss; S1, 25% hair loss; S2, 26–50% hair loss; S3, 51–75% hair loss; S4, 76–99% hair loss; S5, 100% hair loss. Subsequently, the patients were divided into two groups: with localized alopecia areata (S1-S4) and alopecia totalis/universalis (S5). The activity of hair loss was evaluated and defined as: (1) progressive alopecia areata, an increase in the total hair loss of more than 5%; (2) stable, a change in the total hair loss of less than 5%; (3) remitting alopecia areata, a decrease in the total hair loss of more than 5% over the month prior to the laboratory tests [14].

### Biochemical measurements

Venous blood samples were collected after an overnight 12-hour fast. The serum concentrations of lipocalin-2 and visfatin were measured using a commercially available ELISA kit (EIAab, Wuhan, China) according to the manufacturer’s instructions. Moreover, the serum levels of total cholesterol, HDL-cholesterol, triglycerides, glucose, insulin and c-peptide were measured. LDL-cholesterol was calculated in accordance with the following formula: LDL-cholesterol = [triglycerides–HDL-cholesterol–(triglycerides ⁄ 5) [15]. HOMA-IR was calculated as follows: fasting insulin (μIU/mL) × fasting glucose (mg/dL) / 405 [16].

### Statistical analysis

All statistical analyses were carried out with STATISTICA 13.1 (StatSoft, Cracow, Poland). The data were evaluated for the normality of distribution with the Shapiro–Wilk test. Normally distributed variables were expressed as a mean ± standard deviation (SD) while non-normally distributed variables were expressed as a median and interquartile range (IQR). Categorical data were expressed as counts and percentages and were compared using the chi-squared test. Parametric and nonparametric continuous variables were analyzed using the Student’s t-test or the Mann–Whitney U test, respectively. The Spearman’s rank correlation coefficient test was used to assess possible linear associations between two continuous variables. To compare variables according to the disease activity, the Kruskal–Wallis test was used to analyze variables that were not normally distributed and one-way ANOVA for normally distributed variables. The values of *p* < 0.05 were considered statistically significant.

### Ethics

The study protocol conformed to the principles of the World Medical Association’s Declaration of Helsinki and was approved by the Medical University of Warsaw Review Board for Ethics in Human Research (KB/142/2020). Written informed consent was obtained from all the participants of the study.

This project was funded by grant (1M4/3/M/MB/20/20) from the Medical University of Warsaw.

## Results

Fifty-two patients with alopecia areata and 17 control subjects were enrolled in the study. Table 1 shows the demographic characteristics of patients with alopecia areata and healthy controls. As regards study design, both groups of participants did not differ with respect to age, sex distribution and BMI. None of the patients with alopecia areata or control subjects had been diagnosed with glucose or lipid abnormalities before examination.

**Table 1:** Demographic and clinical characteristics of patients with alopecia areata and healthy controls.

Parameter	Patients with alopecia areata (n = 52)	Healthy controls (n = 17)	Statistical significance
Age (years), mean ± SD	39 ± 16	37 ± 11	0.72
Sex (women), n (%)	34 (65%)	12 (71%)	0.69
BMI (kg/m^2^), median (IQR)	23.63 (10.70–27.93)	23.55 (5.78)	0.38
Age of the first episode of alopecia (years), mean (range)	31 (3–70)	NA	
Number of episodes of hair loss, n (range)	3 (1–20)	NA	
Duration of the present episode of alopecia (months), mean (range)	30 (1–300)	NA	
SALT, mean (range)	43 (1–100)	NA	
Severity of hair loss, n (%)		NA	
• S1 - <25%	25 (48%)		
• S2–25–50%	5 (10%)		
• S3–50–75%	7 (13%)		
• S4–75–99%	3 (6%)		
• S5–100%	12 (23%)		
Pattern of hair loss, n (%)		NA	
• Patchy	33 (63%)		
• Ophiasis	3 (6%)		
• Sisaipho	0 (0%)		
• Diffuse	4 (8%)		
• Totalis	3 (6%)		
• Universalis	9 (17%)		
Activity of the disease, n (%)		NA	
Progressive	21 (40.4%)		
Stable	23 (44.2%)		
Remitting	8 (15.4%)		

NA–not applicable

**Fig 1:**
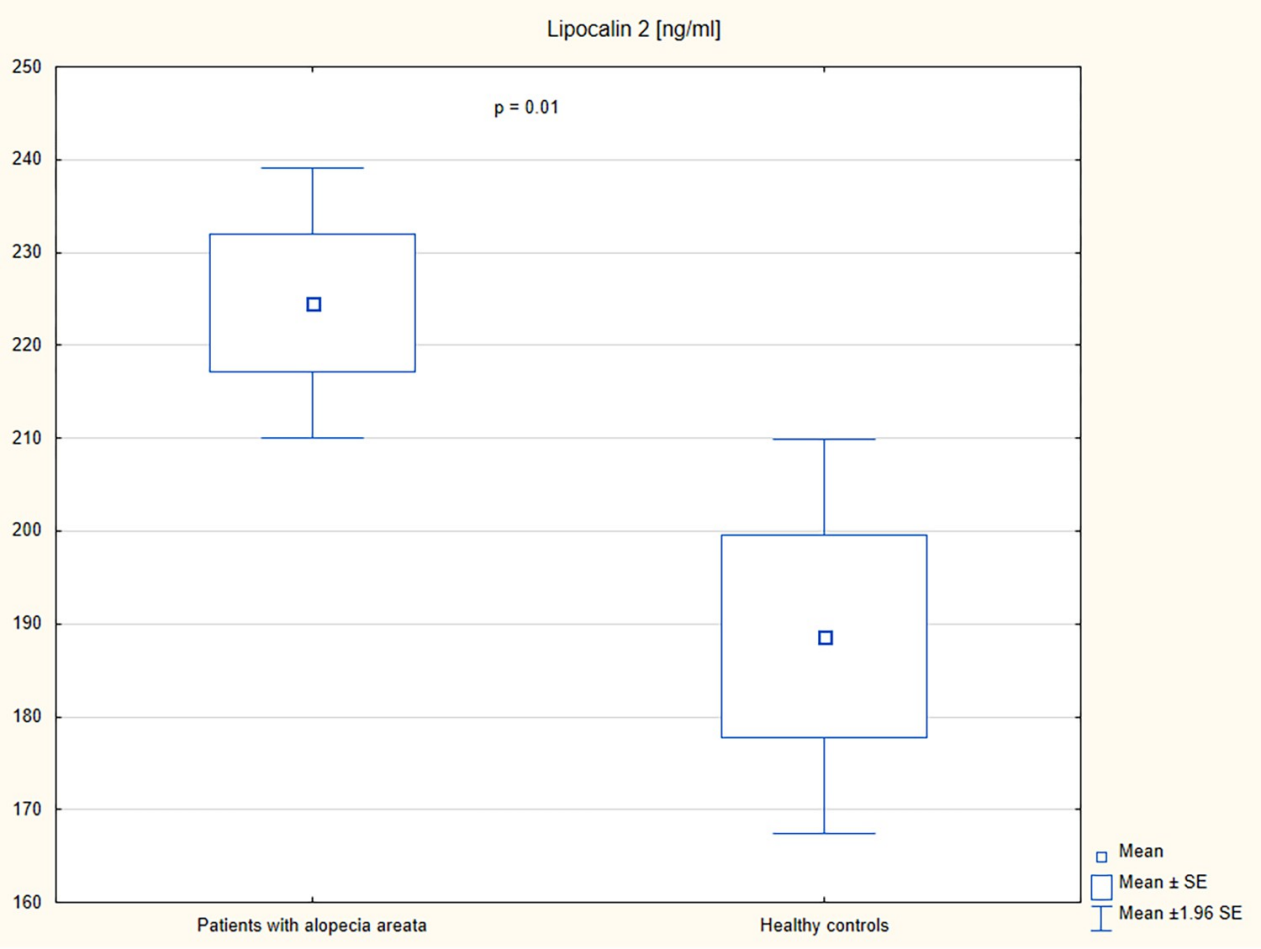
Serum concentrations of lipocalin-2 in patients with alopecia areata and healthy controls.

**Fig 2:**
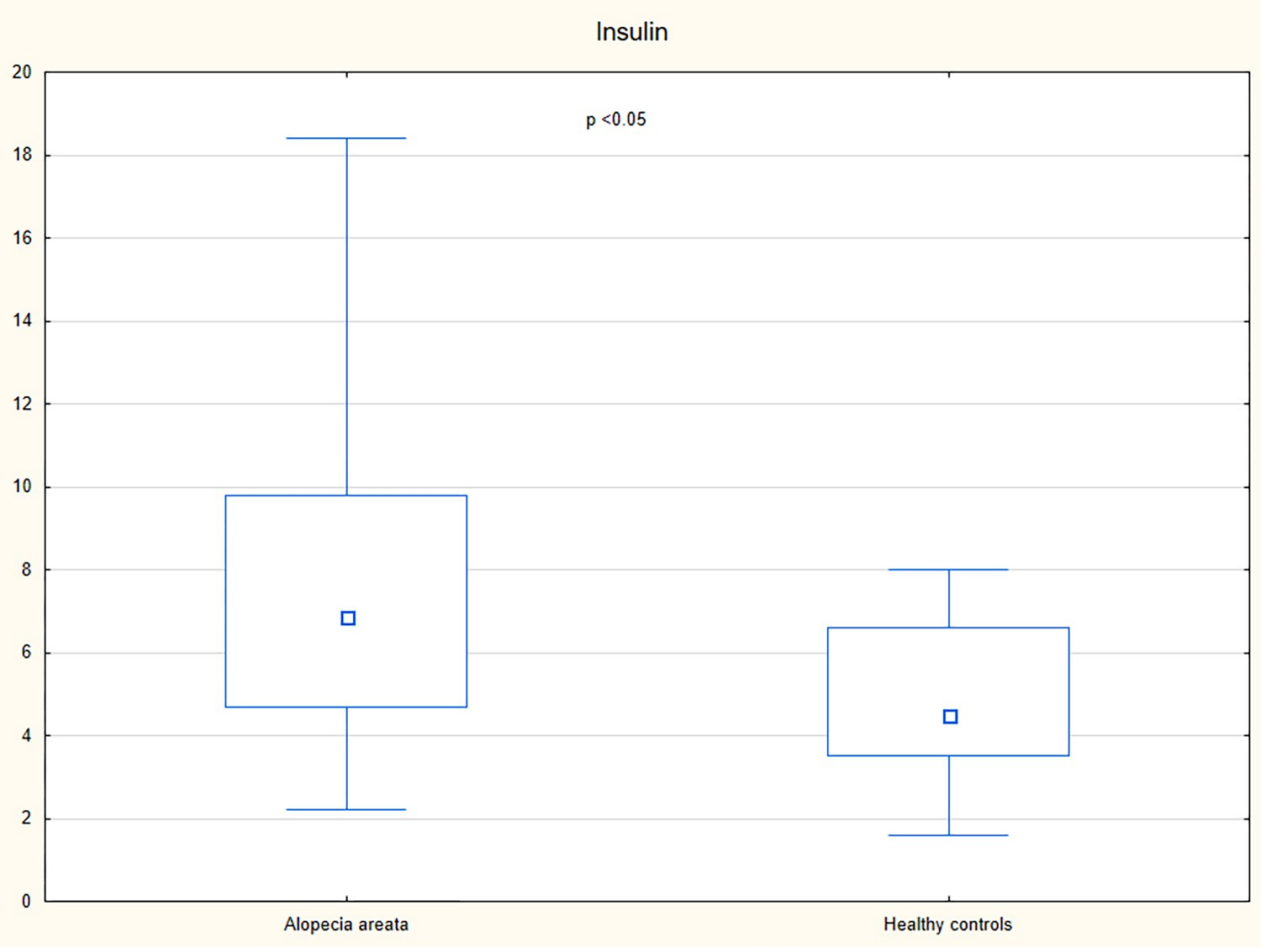
Serum concentrations of insulin in patients with alopecia areata and healthy controls.

**Fig 3:**
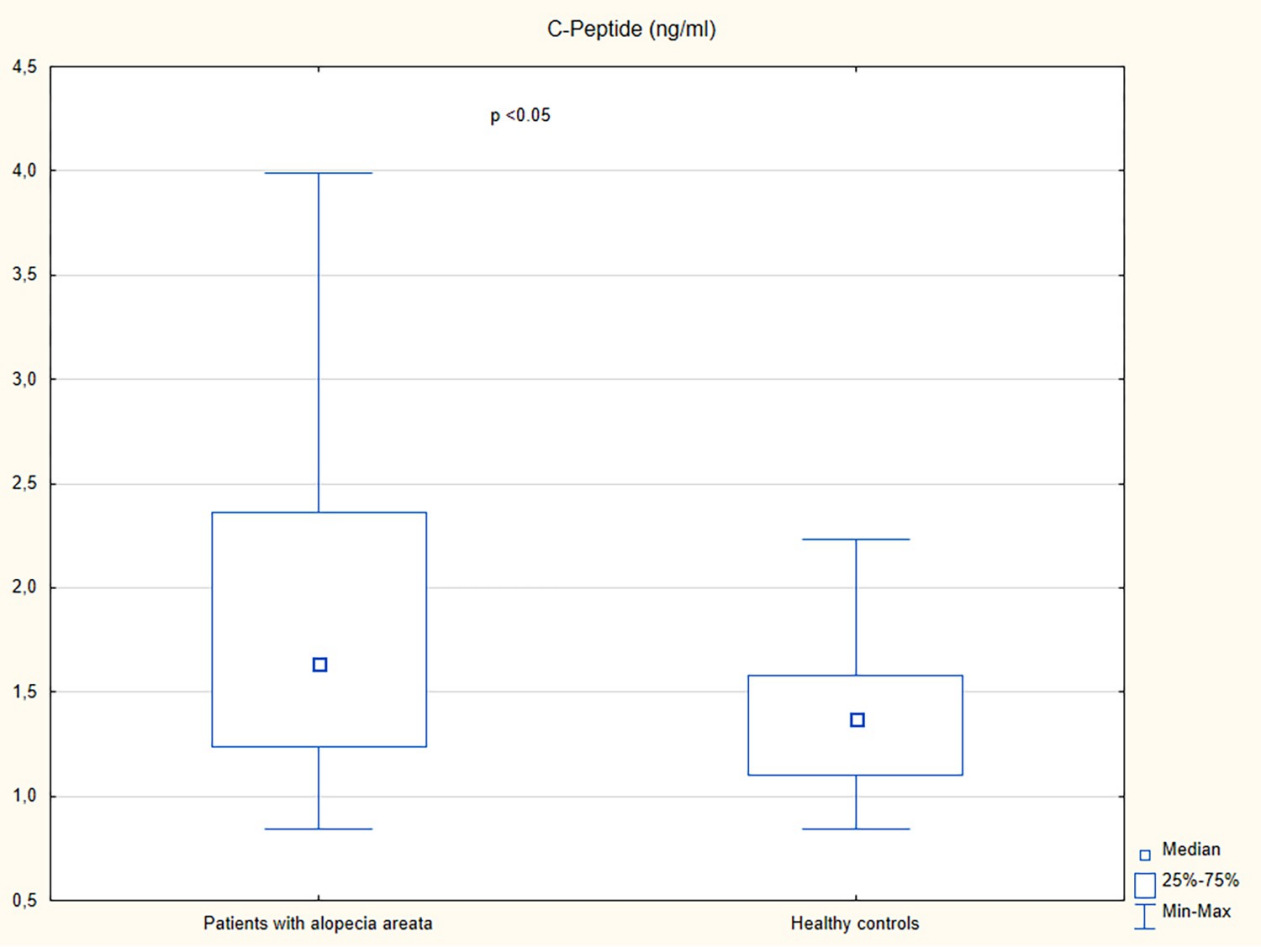
Serum concentrations of c-peptide in patients with alopecia areata and healthy controls.

**Fig 4:**
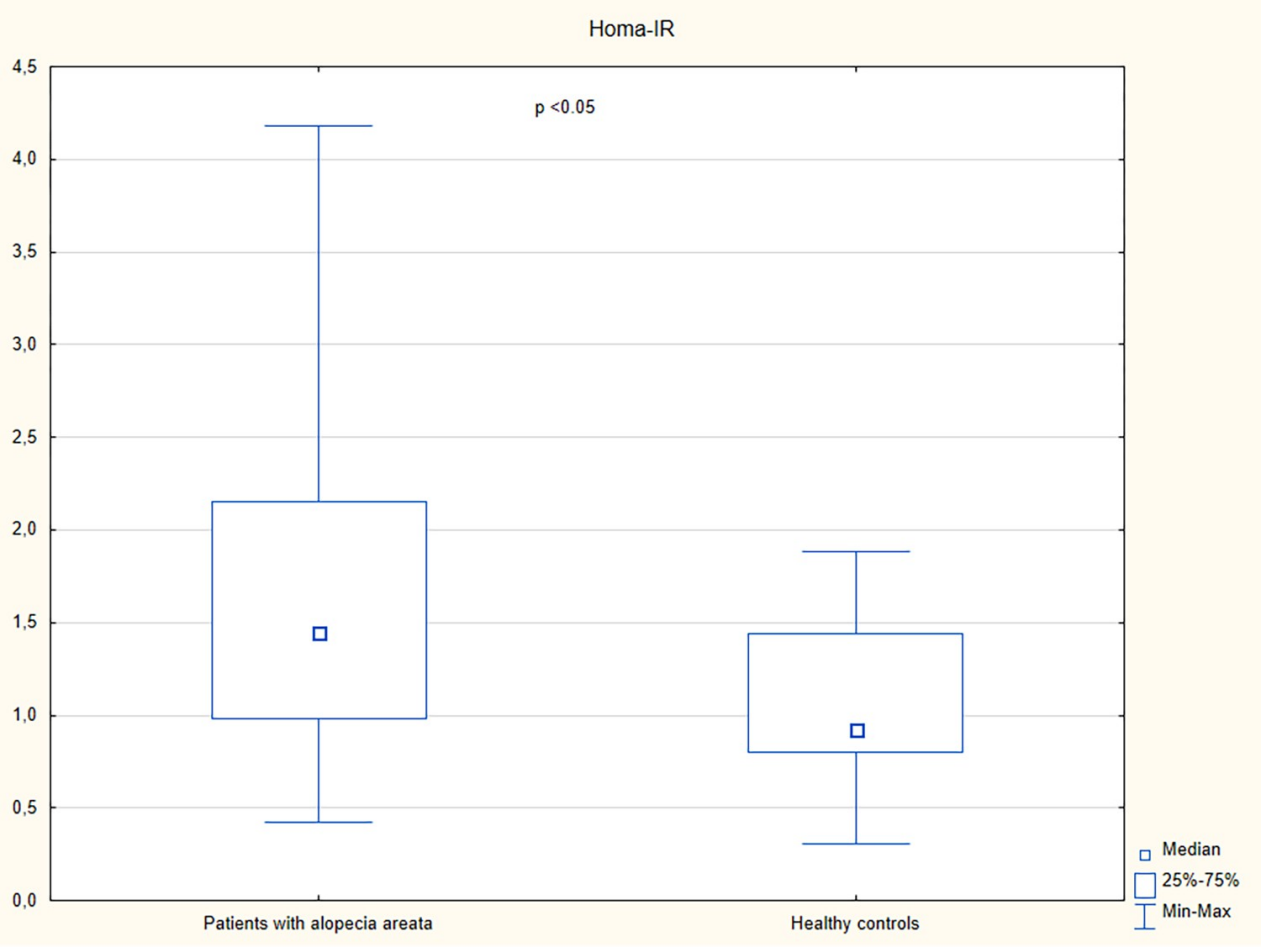
HOMA-IR in patients with alopecia areata and healthy controls.

A higher serum concentration of lipocalin-2 was observed in patients with alopecia areata than in the controls (p = 0.01) (Table 2 and Fig 1). Increased levels of insulin, c-peptide, and HOMA-IR were observed in patients with alopecia areata in comparison with the control group (p<0.05) (Table 2 and Figs 2–4). However, no significant differences were noted in the frequency of the impaired fasting glucose, hyperinsulinemia, insulin resistance, hyperlipidemia, and hypertriglyceridemia in patients with alopecia areata compared to healthy controls (Table 3).

**Table 2:** Laboratory characteristics in patients with alopecia areata and the control group.

Parameter	Patients with alopecia areata (n = 52)	Healthy controls (n = 17)	Statistical significance
Lipocalin-2 (ng/ml), mean ± SD	224.55 ± 53.58	188.64 ± 44.75	0.01
Visfatin (ng/ml), median (IQR)	35.93 (19.72–66.83)	45.71 (19.26–70.66)	0.67
Cholesterol (mg/dl), mean ± SD	190 ± 42	192 ± 26	0.89
LDL-cholesterol (mg/dl), mean ± SD	118 ± 39	121 ± 29	0.80
HDL-cholesterol (mg/dl), mean ± SD	67 ± 120	67 ± 19	0.99
Triglycerides (mg/dl), median (IQR)	95 (65–158)	93 (58–128)	0.28
Glucose (mg/dl), median (IQR)	89 (82–97)	83 (79–90)	0.08
Insulin (μIU/ml), median (IQR)	6.85 (4.7–9.8)	4.5 (3.5–6.6)	<0.05
HOMA-IR, median (IQR)	1.44 (0.98–2.15)	0.92 (0.79–1.44)	<0.05
C-peptide (ng/ml), median (IQR)	1.63 (1.23–2.36)	1.37 (1.1–1.58)	<0.05

SD–standard deviation; IQR–interquartile range

**Table 3:** The frequency of laboratory abnormalities in patients with alopecia areata and healthy controls.

Parameter	Patients with alopecia areata (n = 52)	Healthy controls (n = 17)	Statistical significance
Impaired fasting glucose*, n (%)	10 (19%)	1 (6%)	0.19
Hyperinsulinemia**, n (%)	9 (17%)	0 (0%)	0.67
Insulin resistance***, n (%)	7 (13%)	0 (0%)	0.11
Hyperlipidemia****, n (%)	30 (58%)	13 (76%)	0.16
Hypertriglyceridemia*****, n(%)	14 (27%)	4 (24%)	0.78

* fasting glucose 100–125 mg/dl

** insulin >10.4 μIU/ml

*** HOMA-IR >2.5

**** total cholesterol ≥190 mg/dl and/or LDL-cholesterol ≥115 mg/dl

***** triglycerides ≥150 mg /dl

Table 4 shows the comparison of clinical and laboratory parameters between patients with alopecia totalis / universalis and patients with localized hair loss. Patients with alopecia totalis / universalis were characterized by the increased serum level of triglycerides compared to patients with localized alopecia areata.

**Table 4:** Selected clinical and laboratory parameters in patients with localized alopecia areata and patients with alopecia totalis / universalis.

Parameter	Patients with localized alopecia areata (n = 40)	Patients with alopecia totalis / universalis (n = 12)	Statistical significance
Age (years), mean ± SD	38 ± 16	41 ± 15	0.648
Sex (women), n (%)	29 (73%)	5 (41%)	0.489
BMI (kg/m2), median (IQR)	22.96 (20.62–27.43)	24.55 (22.21–29.47)	0.440
Lipocalin-2 (ng/ml), mean ± SD	223.86 ± 48.22	226.84 ± 71.13	0.868
Visfatin (ng/ml), median (IQR)	38.76 (20.47–67.71)	35.93 (18.38–58.37)	0.514
Cholesterol, mean ± SD	188 ± 39	198 ± 53	0.470
LDL-cholesterol (mg/dl), median (IQR)	112 (91–132)	129 (114–154)	0.185
HDL-cholesterol (mg/dl), median (IQR)	68 (55–82)	62 (39–73)	0.142
Triglycerides (mg/dl), median (IQR)	86 (65–133)	182 (80–215)	<0.05
Glucose, median (IQR)	91 (83–98)	88 (78–94)	0.344
Insulin, median (IQR)	6.95 (4.9–10.1)	5.8 (4.06–7.65)	0.092
HOMA-IR, median (IQR)	1.55 (1.07–2.17)	1.25 (0.87–1.67)	0.120
C-peptide, median (IQR)	1.66 (1.20–2.44)	1.60 (1.33–2.14)	0.922

SD–standard deviation; IQR–interquartile range

Table 5 summarizes the Spearman’s correlation coefficients between selected clinical and laboratory parameters in patients with alopecia areata. The serum concentration of insulin and HOMA-IR correlated with the number of hair loss episodes (r = 0.300, p<0.05 and r = 0.322, p<0.05, respectively). Moreover, a positive correlation occurred between insulin, HOMA-IR and c-peptide with BMI (r = 0.436, p <0.05; r = 0.384, p<0.05 and r = 0.450, p<0.05, respectively).

**Table 5:** Spearman’s correlation coefficients between selected clinical and laboratory parameters in patients with alopecia areata.

Parameter	Age (years)	BM (kg/m^2^)	Disease duration (months)	Age of alopecia areata onset (years)	Number of hair loss episodes (n)	SALT score (%)	Lipocalin-2 (ng/ml	Visfatin (ng/ml)	Insulin (μIU/ml)	HOMA-IR	C-peptide (ng/ml)
Lipocalin-2 (ng/ml)	-0.052	0.178	0.216	-0.170	0.166	0.090	-	0.046	0.088	0.064	0.167
Insulin (μIU/ml)	-0.123	0.436*	-0.028	-0.091	0.300*	-0.007	0.088	-0.069	-	0.957*	0.591*
HOMA-IR	-0.116	0.384*	-0.037	-0.044	0.322*	-0.025	0.064	-0.094	0.957*	-	0.578*
C-peptide (ng/ml)	0.095	0.450*	-0.033	-0.012	0.203	0.233	0.167	0.013	0.591*	0.578*	-

*p <0.05

No correlation between the serum level of lipocalin-2 and fasting glucose, insulin, HOMA-IR or BMI was observed (p>0.05).

Table 6 shows the comparison of clinical and laboratory parameters between patients with progressive, stable and remitting alopecia areata. No significant differences were observed in those patient groups.

**Table 6:** Selected clinical and laboratory parameters in patients with progressive, stable and remitting alopecia areata.

Parameter	Progressive alopecia areata* (n = 21)	Stable alopecia areata** (n = 23)	Remitting alopecia areata*** (n = 8)	Statistical significance
Age (years), mean ± SD	38 ± 14	38 ± 17	45 ± 16	0.536
Sex (women), n (%)	15 (71%)	14 (61%)	5 (63%)	0.749
BMI (kg/m2), median (IQR)	22.46 (20.54–30.82)	24.03 (20.70–27.77)	24.38 (21.97–25.53)	0.952
Lipocalin-2 (ng/ml), median (IQR)	207.2 (173.5–232.8)	235.9 (199.3–280.6)	234.4 (207.4–256.8)	0.128
Visfatin (ng/ml), median (IQR)	60.9 (20.02–66.17)	23.05 (17.94–67.92)	54.21 (23.91–65.81)	0.539
Cholesterol, mean ± SD	185 ± 28	193 ± 51	195 ± 48	0.788
LDL-cholesterol (mg/dl), mean ± SD	116 ± 29	119 ± 43	122 ± 55	0.934
HDL-cholesterol (mg/dl), mean ± SD	65 ± 16	67 ± 23	71 ± 23	0.741
Triglycerides (mg/dl), median (IQR)	95 (63–141)	94 (65–165)	98 (74–177)	0.797
Glucose (mg/dl), median (IQR)	91 (81–99)	88 (78–96)	94 (83–102)	0.416
Insulin, (μIU/ml), median (IQR)	7.2 (5.7–9.2)	6.3 (4.2–9.0)	7.95 (4.6–10.95)	0.323
HOMA-IR, median (IQR)	1.57 (1.24–2.16)	1.27 (0.93–1.92)	1.74 (0.94–2.71)	0.259
C-peptide (ng/ml), median (IQR)	1.78 (1.51–2.1)	1.55 (1.13–2.35)	2.01 (1.33–2.66)	0.689

SD–standard deviation; IQR–interquartile rang

*progressive alopecia areata: An increase in the total hair loss of more than 5%

**stable alopecia areata: A change in the total hair loss of less than 5%

***remitting alopecia areata: A decrease in the total hair loss of more than 5% over the month prior to the laboratory tests

## Discussion

Lipocalin-2, also known as neutrophil gelatinase–associated lipocalin, is a 25-kDa glycoprotein secreted by adipocytes and immune cells such as neutrophils and macrophages [17].

Expression of lipocalin-2 is induced by many proinflammatory cytokines. The level of this adipokine correlates with the level of numerous inflammatory markers such as high sensitivity C-reactive protein [17, 18]. It has been suggested that lipocalin-2 plays an important role in glucose homeostasis and insulin sensitivity. Indeed, an increased serum level of lipocalin-2 in patients with diabetes mellitus compared to healthy controls was described [18]. However, no correlation between the adipokine level and HOMA-IR or glycated hemoglobin was found [17, 18]. Recent studies have demonstrated increased serum levels of lipocalin-2 in inflammatory disorders including psoriasis, atopic dermatitis or rheumatoid arthritis [11, 12]. To date, lipocalin-2 has not been evaluated in alopecia areata. In the present study, an increased serum level of lipocalin-2 was observed in patients with alopecia areata compared to healthy controls (p = 0.01). It is worth to emphasize that in patients with alopecia areata no correlation between lipocalin-2 and fasting glucose, insulin, HOMA-IR or BMI was presented (p>0.05). This observation may suggest that lipocalin-2 is independent marker of alopecia areata not associated with glucose homeostasis or obesity.

Visfatin is a 52 kDa protein composed of 473 amino acids secreted mainly by the adipocytes of visceral adipose tissue [10]. It stimulates the production of pro-inflammatory cytokines (IL-1, IL-6, and TNF-α), which are linked to infectious and inflammatory diseases [8]. The role of visfatin in the regulation of energy homeostasis has been suggested. Visfatin release is promoted by hyperglycemia in healthy humans. Visfatin expression is also increased in obesity, as well as in other states of insulin resistance [8]. Recently, an increased serum level of visfatin has been described in various autoimmune and inflammatory diseases such as rheumatoid arthritis, systemic sclerosis, psoriasis or atopic dermatitis [8–10]. In the present study, similarly to a study conducted by Incel-Uysal et al. [19], no difference was observed in serum visfatin levels between patients with alopecia areata and healthy controls.

It was suggested that alopecia areata, as an autoimmune disease, might be associated with an increased risk of metabolic diseases. Indeed, a cross-sectional study performed by Conic et al. [5], which included 33,130 patients with alopecia areata and 5,246,350 non-alopecia areata controls, revealed that obesity (18.1% vs. 3.0%), hyperlipidemia (19.8% vs. 6.6%), diabetes mellitus (11.4% vs. 7.4%) and metabolic syndrome (1.4% vs. 0.3%) were more commonly reported in patients with alopecia areata compared to the control group. In the present study, impaired fasting glucose was detected in 19% (10/52) of patients with alopecia areata and 6% (1/17) of controls (p<0.19). Hyperinsulinemia and insulin resistance were observed in 17% (9/52) and 13% (7/52) of patients with alopecia areata, respectively, and they were not detected in healthy individuals. However, the differences were not statistically significant (p = 0.67 and p = 0.11, respectively). In spite of having similar fasting blood glucose levels, patients with alopecia areata had higher serum levels of insulin, c-peptide and HOMA-IR in comparison with healthy controls (p<0.05). The observations are similar to studies conducted by Karadag et al. [16] and Shahidi-Dadras et al. [20] who observed the increased serum levels of insulin, c-peptide, and HOMA-IR in patients with alopecia areata compared to control subjects. However, a study conducted by Karadag et al. [16] revealed higher frequency of hyperinsulinemia in patients with alopecia areata (60.8% of patients with alopecia areata and 30.6% of healthy controls; p = 0.05). According to Shahidi-Dadras et al. [20] patients with alopecia totalis/universalis had higher levels of insulin and HOMA-IR compared to those with patchy hair loss (p = 0.01). However, no such differences were detected in the present study. The present analysis showed a positive correlation of insulin and HOMA-IR with the number of alopecia areata episodes. However, positive correlation between these variables and BMI was also presented. Thus, it remains unclear if insulin is associated with higher risk of alopecia areata relapses.

In the present study, similarly to a study conducted by Karadag et al. [16], no significant differences occurred in the concentration of total cholesterol, LDL-cholesterol, HDL-cholesterol and triglycerides between patients with alopecia areata and healthy controls. However, a higher triglyceride level was detected in patients with alopecia totalis or universalis in comparison with individuals with localized alopecia areata (p<0.05).

## Conclusions

The results of the present study suggest that lipocalin-2 and insulin may serve as biomarkers in alopecia areata. Further studies are needed to evaluate the role of insulin as a prognostic factor in alopecia areata.
